# Calorie restriction inhibits ovarian follicle development and follicle loss through activating SIRT1 signaling in mice

**DOI:** 10.1186/s40001-015-0114-8

**Published:** 2015-03-12

**Authors:** Wei-Juan Liu, Xing-Mei Zhang, Na Wang, Xiao-Ling Zhou, Yu-Cai Fu, Li-Li Luo

**Affiliations:** Department of Gynaecology and Obstetrics, The First Affiliated Hospital of Shantou University Medical College, No. 57 Changping Road, Shantou, 515041 China; Laboratory of Cell Senescence, Shantou University Medical College, No. 22 Xinling Road, Shantou, 515041 China

**Keywords:** Calorie restriction, Ovarian development, SIRT1 signaling, SIRT1 activator, Mice

## Abstract

**Background:**

Silent information regulator 2 related enzyme 1 (SIRT1) is one of the key factors in the mechanism of calorie restriction (CR) extending lifespan of animals. The aim of the study is to investigate if CR prolongs ovarian lifespan in mice through activating SIRT1 signaling.

**Methods:**

In the present study, 21 female C57BL/6 mice were divided into three groups: the control (*n* = 7), CR (*n* = 7), and SRT1720 (*n* = 7) groups. After the 26-week treatment, the number of ovarian follicles at each stage was counted, and Western blot was performed.

**Results:**

The number of surviving follicles in ovaries of the SRT1720 group was less than that of the CR group but more than that of the normal control (NC) group. The number of atretic follicles in the ovaries of the SRT1720 group was similar to that of the CR group but less than that of the NC group. The number of primordial follicles in the ovaries of the SRT1720 group was less than that of the CR group but more than that of the NC group. The numbers of primary follicles, secondary follicles, antral follicles, and corpora lutea in the SRT1720 group were similar to those in the CR group. Western blot analysis showed that the expression of SIRT1, SIRT6, FOXO3a, and NRF1 proteins was upregulated, and p53 was downregulated in both the CR group and the SRT1720 group compared to the control group.

**Conclusions:**

Our results indicate that CR inhibits the activation of primordial follicles and development of follicles at different stages, thus preserving the reserve of follicle pool (at least partly) through activating SIRT1 signaling.

## Background

Mammalian females possess a finite number of primordial follicles at birth, following the formation of the primordial follicle pool. Cohorts of these follicles are recruited to begin maturation but the ova do not proliferate, resulting in recruitment continuing until the primordial follicle population becomes depleted. These processes directly affect the number of oocytes available to a female throughout her reproductive life [1]. Once the pool of primordial follicles is depleted, a series of physiological changes known as menopause occur. Inappropriate coordination of these processes contributes to ovarian pathologies, such as premature ovarian failure (POF) and infertility. Therefore, if we can intervene follicle development and follicle loss, the female reproductive life can be extended, the postmenopausal period can be shortened, and female life quality can be enhanced.

Previous studies showed that calorie restriction (CR) is the only non-genetic intervention that has increased the lifespan and delayed age-associated physiological deterioration in species ranging from yeast to mammals [2-4]. Moreover, CR can prolong female reproductive life [5], maintain the follicle reserve [6], and delay ovarian failure of adult rats [7]. However, the molecular mechanism of CR involved in ovarian follicle development and ovarian lifespan is not clear.

Silent information regulator 2 related enzyme 1 (SIRT1) is a dependent histone deacetylase which is closely related to a cell differentiation, senescence, apoptosis, and energy metabolism. Accumulating evidence has demonstrated that SIRT1 is required for CR to mediate lifespan extension in mammals [8,9]. Our previous study showed that CR inhibited the transition from primordial to developing follicles and extended the entire growth phase of a follicle to preserve the reserve of germ cells and suggested that SIRT1 and SIRT6 might be both associated with these CR effects [10]. SIRT1 transgenic mice showed phenotypes resembling CR and delayed sexual maturity, suggesting that SIRT1 may suppress ovarian follicle development and maturation [11]. Therefore, we suppose that SIRT1 may play a key role in CR effects on ovarian follicle development. SRT1720 is a specific SIRT1 activator that has showed health and lifespan benefits in adult mice fed a high-fat diet with a higher dose (100 mg/kg body weight) and a lower dose of SRT1720 (30 mg/kg) [12]. It was also found that SRT1720 could extend lifespan, delay onset of age-related metabolic diseases, and improve general health in mice fed a standard diet [13].

It has been noted that SIRT1 targets are presumably affected by CR. Forkhead box group O (FOXO3a) is an important substrate of SIRT1, and FOXO3a^−/−^ mice showed over-activation of primordial follicles and POF [14]. Sexual maturity is delayed and follicle development is inhibited in FOXO3a transgenic mice [15]. Nuclear respiratory factor 1 (NRF1) is known to regulate mtDNA replication and transcription in various tissues [16] and identified as a nuclear transcription factor that trans-activates the promotors of a number of mitochondrial genes [17]. These include respiratory subunits and factors involved in the replication and transcription of mtDNA. Our previous study demonstrated that CR extended ovarian lifespan by increasing the follicle reserve, and the expression levels of SIRT1, SIRT6, FOXO3a, and NRF1 proteins in the ovaries were also increased [18], suggesting the involvement of SIRT1, SIRT6, FOXO3a, and NRF1 in CR effects on ovarian follicle development. In addition, a recent study revealed that glucose deprivation enhanced the formation of a SIRT1-FOXO3a-NRF1 protein complex on the SIRT6 promoter that is responsible for the induction of SIRT6 expression [19]. p53 is regulated by SIRT1-mediated deacetylation. The expression of p53 protein in the apoptotic granulosa cells of atretic follicles suggests its possible role in atresia [20]. This role of p53 is supported by several experiments [21]: (i) inhibition of p53 expression is associated with a marked reduction in the number of apoptotic granulosa cells and atretic follicles [22]; (ii) overexpression of p53 can induce apoptosis in cAMP-stimulated cells. Moreover, negative p53 patterns may be a favorable prognostic finding showing genome integrity in the replicating follicle cells of women of reproductive age [23].

Based on these data, we suppose that CR may suppress ovarian follicle development and follicle loss by activating SIRT1-FOXO3a-NRF1-SIRT6 signaling and inhibiting some of SIRT1 target molecules such as p53, thus extending the ovarian lifespan of female mice. The present study examined effects of SRT1720 and CR on the follicle development and follicle loss, as well as the expression of key molecules in SIRT1 signaling such as SIRT1, SIRT6, FOXO3a, NRF1, and p53, and discussed the possible mechanisms of regulation.

## Methods

### Animals and treatments

Twenty-one 8-week-old female C57BL/6 mice (body weight :19 ± 1.1 g) were purchased from the Medical Experimental Animal Center of Guangdong Province and housed three or four in stainless steel cages in a room with an ambient temperature of 22°C ± 2°C and a 12-h light: 12-h dark cycle. All animal protocols were approved by the Institutional Animal Care and Use Committee of Shantou University. All the mice were provided a standard rodent chow (20.11% protein, 4.84% lipid, 7.34% crude fiber; total energy 17.3282 MJ/kg) and tap water *ad libitum* for 6 weeks before their division into three groups: the normal control (NC) group, the CR group, and the SRT1720 group. The mice of the NC group were fed *ad libitum* the standard rodent chow. The CR group was subjected to a 30% reduction in the total amount of food that was consumed by the NC group. The SRT1720 group was firstly fed *ad libitum* the standard chow for the first 18 weeks and then given the standard chow containing SRT1720 (50 mg/kg B.W.) every other day for the last 8 weeks.

The food intake of the mice was measured once every month. According to the NC group, the food intake of the CR group was timely modulated. Body weight was measured once a month. Estrous cycle was examined by vaginal smears once a month over more than 16 days. After 26 weeks, mice were anesthetized with pentobarbital sodium at 40 mg/kg body weight, and sacrificed by cervical dislocation. Mouse perirenal fat was isolated and weighed and expressed as visceral fat index. Both ovaries of each mouse were removed and weighed. One was stored at −80°C for Western blot analysis. The other one was used for histological analysis.

### Estrous cycle analysis

Vaginal cytology was assessed daily between 9:00 AM and 10:00 AM before and until the end of the treatment to determine the estrous cycle of each mouse. Vaginal cells were collected via a sterile cotton swab saturated with normal saline (NaCl, 0.9%), and then placed on a clean glass slide to form well-proportioned tracks of smears. The smears were stained with Giemsa and examined under light microscope. Stages were assessed based on vaginal cytology [24]. A proestrus smear consisted of predominantly nucleated cornified cells; an estrus smear consisted primarily of anucleated cornified cells; a metestrus smear consisted of an equal proportion of leukocytes, cornified, and nucleated epithelial cells; and a diestrus smear consisted of primarily leukocytes. A 4- to 5-day estrous cycle was determined to be a regular cycle, and a cycle duration of >5 days or <4 days was considered to be an irregular cycle [25].

### Preparation of ovarian sections

One of the two ovaries from each mouse was fixed in 4% paraformaldehyde at 4°C overnight, then dehydrated using a series of ethanol washes, cleared in xylene, and embedded in paraffin. Ovarian sections of 4 μm thickness were prepared for hematoxylin and eosin (HE) staining.

### HE staining and follicle classification

The sections were deparaffinized in xylene, hydrated using a series of ethanol washes, and stained with HE using standard protocols. Sections were mounted using Canada balsam and observed under a light microscope. Every section from each ovary was used for follicle counting, and then correcting for double counting. Follicles were classified according to a previous study [26] as follows: primordial follicle (an oocyte surrounded by one layer of flattened granulosa cells), primary follicle (an oocyte surrounded by one layer of cuboidal granulosa cells), secondary follicle (two or three layers of cuboidal granulosa cells with no antral space), and antral follicle (more than four layers of granulosa cells with one or more independent antral spaces or with a cumulus granulosa cell layer). In some cases, antral follicles showed no antral space in cross section analysis but were considered antral if they contained ≥5 granulosa cell layers. Follicles were classified as either healthy (intact basal-lamina, oocyte with no more than three cytoplasmic vacuoles, intact germinal vesicle and nucleolus) or atretic (apoptotic). Antral follicles were considered atretic if they contained at least 20 apoptotic granulosa cells (defined by the appearance of apoptotic bodies in the granulosa cell layer), disorganized granulosa cells, a degenerating oocyte, or a fragmentation of the oocyte nucleus [27].

### Western blot analysis

Ovarian tissue samples were homogenized in PBS with a Teflon-glass homogenizer (Thomas Scientific, Swedesboro, NJ, USA) on ice and sonicated. Protein concentrations were determined by BCA protein assay (TIANGEN BIOTECH, Beijing, China). The protein samples were separated by SDS-PAGE and transferred onto nitrocellulose membranes (BioTrace™ NT, Port Washington, NY, USA). The membranes were blocked in 5% nonfat dry milk in tris-buffered-saline with tween 20 (TBST) for 1 h and incubated with a primary antibody against SIRT1, SIRT6, and FOXO3a (1:200 dilution, Santa Cruz Biotechnology, CA, USA), NRF1 (1:400 dilution, Santa Cruz Biotechnology, CA, USA), p53 (1:600 dilution, Santa Cruz Biotechnology, CA, USA) or β-action (1:1,000 dilution, Santa Cruz Biotechnology, CA, USA) over-night at 4°C, followed by the incubation with a secondary (horseradish peroxidase-conjugated) anti-rabbit or anti-mouse antibody (1:5,000 diluted) at room temperature for 1 h. Bands were visualized with a chemiluminescence reagent (Thermo Fisher Scientific, Waltham, MA, USA). Band intensities were analyzed using the Quantity One software (Bio-Rad Laboratories Pty. Ltd., Hercules, CA, USA). β-actin was used as a loading control.

### Statistical analysis

All results are expressed as the means ± SEM (*n* = 7 in every group) and analyzed by the SPSS 17.0 software. A one-way ANOVA was used to compare the data among groups. A *P* value less than 0.05 was considered as statistical significance (*P* < 0.05).

## Results

All mice were alive at the end of the 26-week treatment, and no superficial abnormalities or tumors were found in the abdomen and other parts of the body.

### Energy intake, body weight, and visceral fat

**Figure 1:**
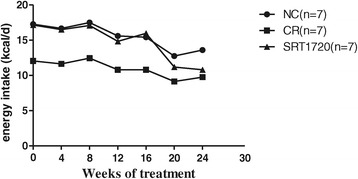
**Mouse energy intake (the mice in the SRT1720 group were given the standard chow containing SRT1720 (50 mg/kg B.W.) every other day for the last 8 weeks).** CR, calorie restriction; NC, normal control.

The pattern of energy intake of the SRT1720 group was as similar as that of the NC group before SRT1720 treatment. After SRT1720 treatment, the energy intake of the SRT1720 group gradually decreased compared to the NC group. At the end of treatment, the energy intake of the SRT1720 group (10.80 ± 0.18 kcal/day) was comparable to that of the CR group (9.5 kcal/day, *P* > 0.05) but lower than that of the NC group (13.58 ± 0.25 kcal/day, *P* < 0.05) (Figure 1).

**Figure 2:**
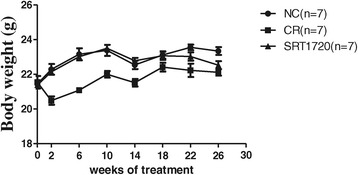
**Changes in the body weight of mice (the mice in the SRT1720 group were given the standard chow containing SRT1720 (50 mg/kg B.W.) every other day for the last 8 weeks).** CR, calorie restriction; NC, normal control.

The body weight among groups was comparable to each other at the beginning of the treatment (*P* > 0.05). The body weight in the NC group and the SRT1720 group increased from the beginning to the end of the treatment (21.45 ± 0.11 vs 23.34 ± 0.22 g, *P* < 0.05; 21.39 ± 0.20 vs 22.53 ± 0.23 g, *P* < 0.05). The body weight in the CR group was similar from the beginning to the end (21.54 ± 0.36 vs 22.11 ± 0.19 g, *P* > 0.05). The pattern of body weight of the SRT1720 group was as similar as that of the NC group before SRT1720 treatment. After SRT1720 treatment, body weight of the SRT1720 group decreased compared to the NC group. At the end of the experiment, the body weight of the SRT1720 mice was similar to that of the CR mice (22.53 ± 0.23 vs 22.11 ± 0.19 g, *P* > 0.05). But the body weight of the SRT1720 mice or the CR mice was lower than that of the NC mice (22.53 ± 0.23 vs 23.34 ± 0.22 g, *P* < 0.05; 22.11 ± 0.19 vs 23.34 ± 0.22 g, *P* < 0.05) (Figure 2).

The visceral fat weight in the SRT1720 group was similar to that in the CR group (0.36 ± 0.01 vs 0.29 ± 0.01 g, *P* > 0.05). But the visceral fat weight in the SRT1720 group or the CR group decreased compared to that of the NC group (0.36 ± 0.01 vs 0.53 ± 0.03 g, *P* < 0.05; 0.29 ± 0.01 vs 0.53 ± 0.03 g, *P* < 0.05).

### Ovarian weight

**Figure 3:**
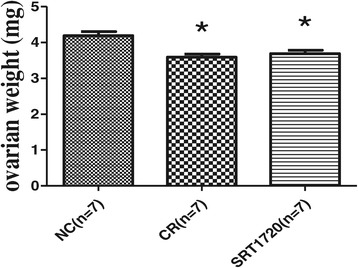
**Comparison of ovarian weight among groups.** Values were represented as means ± SEM. *P < 0.05 vs the control group. CR, calorie restriction; NC, normal control.

The ovarian weight in the SRT1720 group was similar to that in the CR group (3.69 ± 0.10 vs 3.59 ± 0.09 mg, *P* > 0.05). But the ovarian weight in the SRT1720 group or the CR group was lower than that of the NC group (3.69 ± 0.10 vs 4.19 ± 0.11 mg, *P* < 0.05; 3.59 ± 0.09 vs 4.19 ± 0.11 mg, *P* < 0.05) (Figure 3).

### Estrous cycle

We examined the estrous cycles of all mice before the treatment and found that all of them represented regularly estrous cycles (4 to 5 days). During the experiment, mice in the CR group gradually lost their regular estrous cycles. At the end of the experiment, six (85.71%) mice in the CR group showed irregular estrous cycle, and all of them displayed estrous cycles >5 days in intermittent. All the NC mice (100%) exhibited regular estrous cycles during the first 5 months of treatment, but 1 (14.29%) mouse showed irregular estrous cycle at the sixth month of treatment. Before SRT1720 treatment, all (100%) mice in the SRT1720 group also displayed regular estrous cycles. During SRT1720 treatment, 5 (71.43%) mice in the SRT1720 group showed irregular estrous cycle (Table 1).

**Table 1:** **Percentage of mice at different stages of the estrous cycle groups**

**Age and estrous cycle stage**	**NC (** ***n*** **= 7) %**	**CR (** ***n*** **= 7) %**	**SRT1720 (** ***n*** **= 7) %**
4 months			
Regular cycling	7	5 (71.43%)	7
Irregular cycling	0	2 (28.57%)	0
5 months			
Regular cycling	7	4 (57.14%)	7
Irregular cycling	0	3 (42.86%)	0
6 months			
Regular cycling	7	3 (42.86%)	7
Irregular cycling	0	4 (57.14%)	0
7 months			
Regular cycling	7	3 (42.86%)	7
Irregular cycling	0	4 (57.14%)	0
8 months			
Regular cycling	7	2 (28.57%)	2 (28.57%)
Irregular cycling	0	5 (71.43%)	5 (71.43%)
9 months			
Regular cycling	6 (85.71%)	1 (14.29%)	2 (28.57%)
Irregular cycling	1 (14.29%)	6 (85.71%)	5 (71.43%)

Note: The SRT1720 treatment started at the beginning of eighth month of age and stopped at the end of ninth month of age. CR, calorie restriction; NC, normal control.

### Effects of SRT1720 treatment on follicle development and follicle loss

#### Comparison of the total number of follicles, surviving follicles, and atretic follicles among groups

**Figure 4:**
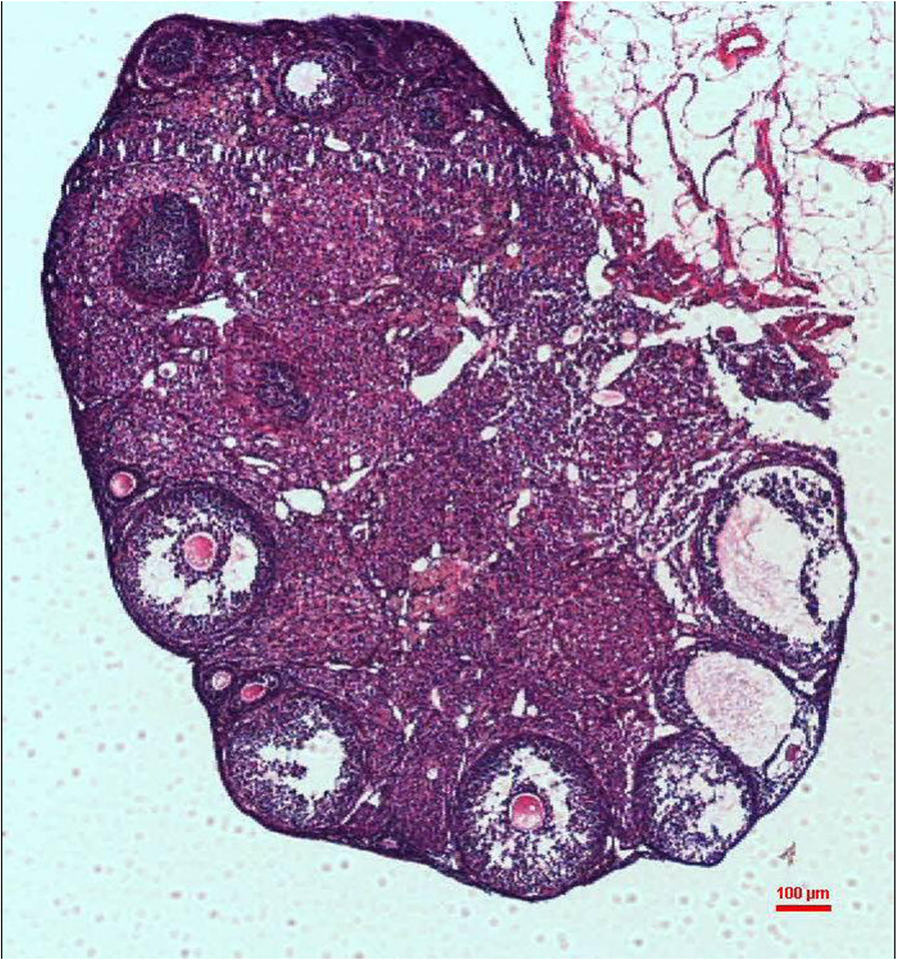
**A representative section of mouse ovary stained by HE.**

**Figure 5:**
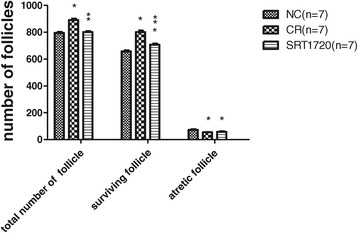
**Comparison of the total number of follicles, survival follicles, and atretic follicles among groups.** *P < 0.05 vs the NC group. **P < 0.05 vs the CR group. CR, calorie restriction; NC, normal control.

HE staining results showed that adult mouse ovaries were mainly composed of surviving follicles (primordial, primary, secondary, and antral follicles), corpora lutea, and atretic follicles (Figure 4). The total number of follicles was defined as the sum of the number of all stages of follicles in one ovary. The total number of follicles in the SRT1720 group was comparable to that in the NC group (802 ± 21 vs 794 ± 19, *P* > 0.05), either of them was less than that in the CR group (802 ± 21 or 794 ± 19 vs 891 ± 29, *P* < 0.05). The number of surviving follicles in the ovaries of the SRT1720 group was less than that of the CR group (707 ± 22 vs 802 ± 27, *P* < 0.05) but more than that of the NC group (707 ± 22 vs 658 ± 18, *P* < 0.05). The number of atretic follicles in ovaries of the SRT1720 group was similar to that of the CR group (59 ± 10 vs 54 ± 7, *P* > 0.05) but less than that of the NC group (59 ± 10 vs 73 ± 10, *P* < 0.05) (Figure 5).

#### Comparison of the number of follicles at different stages among groups

**Figure 6:**
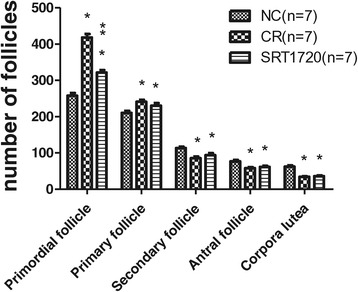
**The distribution of follicles at different stages in the control group, CR group, and SRT1720 group.** *P < 0.05 vs the NC group. **P < 0.05 vs the CR group. CR, calorie restriction; NC, normal control.

The number of primordial follicles in the ovaries of the SRT1720 group was less than that of the CR group (321 ± 19 vs 418 ± 26, *P* < 0.05) but more than that of the NC group (321 ± 19 vs 258 ± 20, *P* < 0.05). The number of primary follicles in the ovaries of the SRT1720 group was similar to that of the CR group (230 ± 19 vs 241 ± 15, *P* > 0.05) but more than that of the NC group (230 ± 19 vs 210 ± 16, *P* < 0.05). The number of secondary follicles in the ovaries of the SRT1720 group was similar to that of the CR group (94 ± 14 vs 85 ± 12, *P* > 0.05) but less than that of the NC group (94 ± 14 vs 114 ± 10, *P* < 0.05). The number of antral follicles in the SRT1720 group was similar to that of the CR group (62 ± 7 vs 58 ± 9, *P* > 0.05) but less than that of the NC group (62 ± 7 vs 77 ± 9, *P* < 0.05). The number of corpora lutea in the ovaries of the SRT1720 group was similar to that of the CR group (36 ± 6 vs 34 ± 3, *P* > 0.05) but less than that of the NC group (36 ± 6 vs 62 ± 10, *P* < 0.05) (Figure 6).

### SIRT1 signaling was enhanced in the ovaries of the SRT1720 group and the CR group

**Figure 7:**
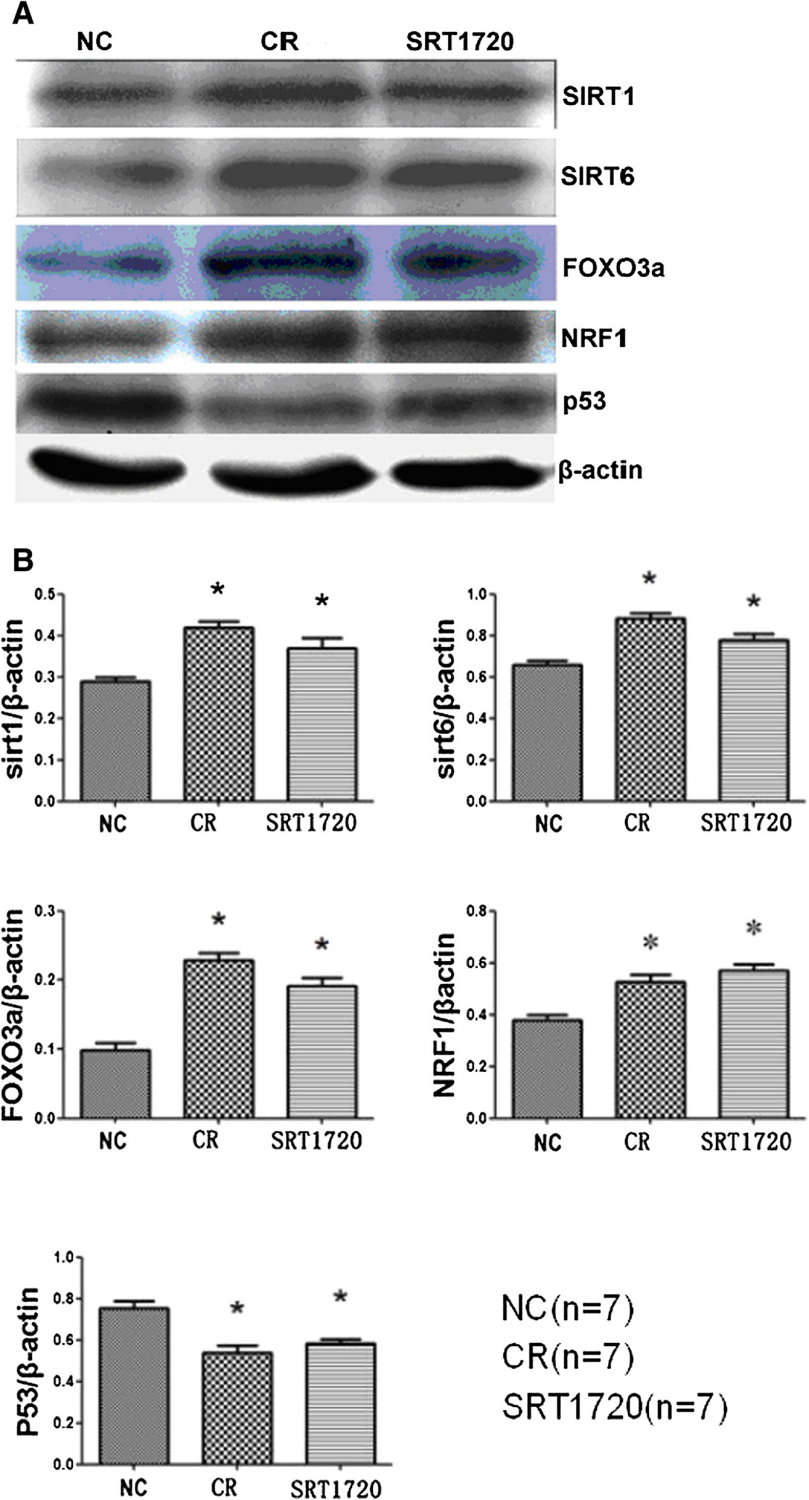
**The protein levels of SIRT1, SIRT6, FOXO3a, NRF-1, and p53 in mouse ovaries. (A)** Western blot results. **(B)** Plot of protein expression. β-Actin was used as internal control. Data are expressed as means ± SEM. *P < 0.05 vs the NC group. CR, calorie restriction; FOXO3a, forkhead box group O; NC, normal control; NRF1, nuclear respiratory factor 1; SIRT1, silent information regulator 2 related enzyme 1.

Our Western blot results showed that the protein expression of SIRT1, SIRT6, NRF1, and FOXO3a in the CR group and the SRT1720 group increased compared to the NC group. However, p53 decreased in the CR group and the SRT1720 group compared to the NC group (*P* < 0.05). The expression levels of SIRT1, SIRT6, NRF1, FOXO3a, and p53 proteins in the SRT1720 group were similar to those in the CR group (*P* > 0.05) (Figure 7).

## Discussion

Menopause is defined as the permanent cessation of menses and irreversible termination of fertility, because of the gradual depletion of oocytes. It leads to consequences that include osteoporosis, obesity, sexual dysfunction, depression, anxiety, and a higher incidence of Alzheimer’s disease and cardiovascular disease [28,29]. Increased life expectancy leads to increased age-associated health issues in both sexes. Women, who generally live longer than men, will spend nearly a third of their lifetime in postmenopause. How to maintain the reproductive health and improve the ovarian lifespan has currently been an area of great interest.

In this study, we found that the body weight and visceral fat in adult female mice 8 weeks after SRT1720 treatment were similar to those in the CR group but lower than those in the NC group. Moreover, SRT1720 treatment could decrease mouse intake. As CR treatment, SRT1720 could decrease mouse intake and body weight. It has been established that the female reproductive aging is associated with a reduced ovarian follicle reserve and gradual declines in regular estrous cyclicity at middle age [30,31]. Hence, we examined the status of estrous cycle among the groups. We found that all the mice displayed regular estrous cycle at the beginning of the experiment. During the experiment, the estrous cycle of mice in the CR group gradually became irregular. At the end, 14.29% NC mice represented irregularly. The incidence of irregular estrous cycle was 85.71% in the CR group and 71.43% in the SRT1720 group. The most abnormal CR mice and SRT1720 mice displayed estrous cycles >5 days. We supposed that irregular estrous cycle of the CR mice and the SRT1720 mice resulted from insufficient estrogen secreted by fewer mature follicles. This is in agreement with the following follicle count results. Follicle count results showed that the number of surviving follicles in ovaries of the SRT1720 group was less than that of the CR group but more than that in the NC group, and the number of atretic follicles was fewer than that of the NC group. This indicated that both CR and SRT1720 treatment could inhibit follicle development and follicle loss and promote follicle reserve. The number of primordial follicles in the SRT1720 group was more than that of the NC group though it was less than that of the CR group. However, the numbers of primary follicles, secondary follicles, antral follicles, and corpora lutea in the SRT1720 group were similar to those in the CR group. These suggest that both SRT1720 and CR may inhibit the activation of primordial follicles, follicle development, follicle maturation, and ovulation. Because of fewer larger follicles and corpora lutea, the volume and weight of the ovaries in the SRT1720 group or the CR group were smaller than those in the NC group.

To explore whether SIRT1 signaling was involved in the ovarian follicle development, we examined the expression of SIRT1 and related factors by Western blot. Kim *et al*. [19] recently reported that SIRT1 is involved in maintaining SIRT6 expression and is required for SIRT6 induction during fasting. The authors further found that there are NRF-1 binding sites in the SIRT6 promoter, and its expression was regulated by CR. In addition, their study showed that FOXO3a was also involved in the regulation of SIRT6 expression, and the downregulation of FOXO3a prevented SIRT6 from responding to the induction of CR and SIRT1, suggesting that SIRT1, FOXO3a, and NRF-1 may form a complex on the SIRT6 promoter, which could be enhanced by glucose deprivation. Therefore, we examined the effect of CR and SRT1720 on SIRT1, FOXO3a, NRF1, and SIRT6 protein expression. Our results showed that both CR and SRT1720 could upregulate SIRT1, FOXO3a, NRF1, and SIRT6 protein expression in the ovary.

p53 is a tumor suppressor gene that is a positive regulator of apoptosis in its native form (wild-type). DNA damage causes a rapid increase in the total concentration of p53 protein by increased gene transcription and the stabilization of normally rapidly degraded p53 protein which leads to the suppression of cell growth and p53-mediated cell-cycle arrest [32]. The expression of p53 protein in the apoptotic granulosa cells of atretic follicles suggests its possible role in atresia [20]. Moreover, p53 is regulated by SIRT1-mediated deacetylation. SIRT1 protein regulates p53 acetylation and p53-dependent apoptosis [33]. In addition, Ghafari *et al*. showed that p53 gene is involved in the regulation and selection of oocytes at checkpoints, such that oocytes that would otherwise be lost may persist when p53 is absent or reduced [34]. These data indicate that p53 is associated with follicle atresia. Therefore, we also examined the effect of CR and SRT1720 on p53 protein expression in the ovary. The results showed that both CR and SRT1720 could inhibit p53 protein expression in the ovaries, which is probably due to the activation of SIRT1.

## Conclusions

Taken together, our present study indicates that CR inhibits the activation of primordial follicles and development of follicles at different stages, thus preserving the reserve of follicle pool and prolonging the ovarian lifespan (at least partly) through activating SIRT1 signaling. We may use SIRT1 specific activator (SRT1720) to imitate CR signal. However, pharmacological application of SRT1720 will be further investigated in the future study.

## Abbreviations

CR: calorie restriction
FOXO3a: forkhead box group O
HE: hematoxylin and eosin
NC: normal control
NRF1: nuclear respiratory factor 1
PAGE: polyacryl amide gel electrophoresis
PBS: phosphate buffer solution
PG: pre-granular cell
POF: premature ovarian failure
SEM: standard error of mean
SDS: sodium dodecyl sulfate
SIRT1: silent information regulator 2 related enzyme 1
SIRT6: silent information regulator 2 related enzyme 6
SPSS: Statistical Package for the Social Sciences
TBST: tris-buffered-saline with tween

## References

[1] Skinner MK (2005). Regulation of primordial follicle assembly and development. Hum Reprod Update.

[2] Canto C, Auwerx J (2009). Caloric restriction, SIRT1 and longevity. Trends Endocrinol Metab.

[3] Masoro EJ (2000). Caloric restriction and aging: an update. Exp Gerontol.

[4] Sohal RS, Weindruch R (1996). Oxidative stress, caloric restriction, and aging. Science.

[5] Osborne TB, Mendel LB, Ferry EL (1917). The effect of retardation of growth upon the breeding period and duration of life of rats. Science.

[6] Nelson JF, Gosden RG, Felicio LS (1985). Effect of dietary restriction on estrous cyclicity and follicular reserves in aging C57BL/6 J mice. Biol Reprod.

[7] Selesniemi K, Lee HJ, Tilly JL (2008). Moderate caloric restriction initiated in rodents during adulthood sustains function of the female reproductive axis into advanced chronological age. Aging Cell.

[8] Boily G, Seifert EL, Bevilacqua L, He XH, Sabourin G, Estey C (2008). SIRT1 regulates energy metabolism and response to caloric restriction in mice. PLoS One.

[9] Chen D, Steele AD, Lindquist S, Guarente L (2005). Increase in activity during calorie restriction requires SIRT1. Science.

[10] Luo LL, Chen XC, Fu YC, Xu JJ, Li L, Lin XH (2012). The effects of caloric restriction and a high-fat diet on ovarian lifespan and the expression of SIRT1 and SIRT6 proteins in rats. Aging Clin Exp Res.

[11] Bordone L, Cohen D, Robinson A, Motta MC, van Veen E, Czopik A (2007). SIRT1 transgenic mice show phenotypes resembling calorie restriction. Aging Cell.

[12] Minor RK, Baur JA, Gomes AP, Ward TM, Csiszar A, Mercken EM (2011). SRT1720 improves survival and healthspan of obese mice. Sci Rep.

[13] Mitchell SJ, Martin-Montalvo A, Mercken EM, Palacios HH, Ward TM, Abulwerdi G (2014). The SIRT1 activator SRT1720 extends lifespan and improves health of mice fed a standard diet. Cell Rep.

[14] Castrillon DH, Miao L, Kollipara R, Horner JW, DePinho RA (2003). Suppression of ovarian follicle activation in mice by the transcription factor FOXO3a. Science.

[15] Liu L, Rajareddy S, Reddy P, Du C, Jagarlamudi K, Shen Y (2007). Infertility caused by retardation of follicular development in mice with oocyte-specific expression of FOXO3a. Development.

[16] Larsson NG, Oldfors A, Holme E, Clayton DA (1994). Low levels of mitochondrial transcription factor A in mitochondrial DNA depletion. Biochem Biophys Res Commun.

[17] Evans MJ, Scarpulla RC (1990). NRF-1: a trans-activator of nuclear-encoded respiratory genes in animal cells. Genes Dev.

[18] Wang N, Luo LL, Xu JJ, Xu MY, Zhang XM, Zhou XL (2013). Obesity accelerates ovarian follicle development and follicle loss in rats. Metabolism.

[19] Kim HS, Xiao C, Wang RH, Lahusen T, Xu X, Vassilopoulos A (2010). Hepatic-specific disruption of SIRT6 in mice results in fatty liver formation due to enhanced glycolysis and triglyceride synthesis. Cell Metab.

[20] Kim JM, Yoon YD, Tsang BK (1999). Involvement of the Fas/Fas ligand system in p53-mediated granulosa cell apoptosis during follicular development and atresia. Endocrinology.

[21] Hussein MR (2005). Apoptosis in the ovary: molecular mechanisms. Hum Reprod Update.

[22] Tilly JL, Tilly KI, Kenton ML, Johnson AL (1995). Expression of members of the bcl-2 gene family in the immature rat ovary: equine chorionic gonadotropin-mediated inhibition of granulosa cell apoptosis is associated with decreased bax and constitutive bcl-2 and bcl-xlong messenger ribonucleic acid levels. Endocrinology.

[23] Depalo R, Nappi L, Loverro G, Bettocchi S, Caruso ML, Valentini AM (2003). Evidence of apoptosis in human primordial and primary follicles. Hum Reprod.

[24] Hiney JK, Srivastava V, Nyberg CL, Ojeda SR, Dees WL (1996). Insulin-like growth factor I of peripheral origin acts centrally to accelerate the initiation of female puberty. Endocrinology.

[25] Schedin P, Mitrenga T, Kaeck M (2000). Estrous cycle regulation of mammary epithelial cell proliferation, differentiation, and death in the Sprague-Dawley rat: a model for investigating the role of estrous cycling in mammary carcinogenesis. J Mammary Gland Biol Neoplasia.

[26] Luo LL, Huang J, Fu YC, Xu JJ, Qian YS (2008). Effects of tea polyphenols on ovarian development in rats. J Endocrinol Invest.

[27] Juliani CC, Silva-Zacarin EC, Santos DC, Boer PA (2008). Effects of atrazine on female Wistar rats: morphological alterations in ovarian follicles and immunocytoch emical labeling of 90 kDa heat shock protein. Micron.

[28] Wise PM, Smith MJ, Dubal DB, Wilson ME, Krajnak KM, Rosewell KL (1999). Neuroendocrine influences and repercussions of the menopause. Endocr Rev.

[29] Lamberts SW, van den Beld AW, van der Lely AJ (1997). The endocrinology of aging. Science.

[30] Anzalone CR, Hong LS, Lu JK, LaPolt PS (2001). Influences of age and ovarian follicular reserve on estrous cycle patterns, ovulation, and hormone secretion in the Long-Evans rat. Biol Reprod.

[31] Lu JK, Anzalone CR, LaPolt PS (1994). Relation of neuroendocrine function to reproductive decline during aging in the female rat. Neurobiol Aging.

[32] Moallem SA, Hales BF (1998). The role of p53 and cell death by apoptosis and necrosis in 4-hydroperoxycyclophosphamide-induced limb malformations. Development.

[33] Cheng HL, Mostoslavsky R, Saito S, Manis JP, Gu Y, Patel P (2003). Developmental defects and p53 hyperacetylation in Sir2 homolog (SIRT1)-deficient mice. Proc Natl Acad Sci U S A.

[34] Ghafari F, Pelengaris S, Walters E, Hartshorne GM (2009). Influence of p53 and genetic background on prenatal oogenesis and oocyte attrition in mice. Hum Reprod.

